# Analysis of Arterial Blood Gas Values Based on Storage Time Since Sampling: An Observational Study

**DOI:** 10.3390/nursrep11030048

**Published:** 2021-07-08

**Authors:** Alejandro Montero-Salinas, Marta Pérez-Ramos, Fernando Toba-Alonso, Leticia Quintana-DelRío, Jorge Suanzes-Hernández, María Sobrido-Prieto, Santiago Martínez-Isasi

**Affiliations:** 1 Emergency Unit, Biomedical Research Institute of A Coruña (INIBIC), Complexo Hospitalario Universitario de A Coruña (CHUAC), Sergas, Universidade da Coruña (UDC), 15001 A Coruña, Spain; monteroenfermero@gmail.com (A.M.-S.); marta.perez.ramos@sergas.es (M.P.-R.); ftobalo2@gmail.com (F.T.-A.); Leticia.Quintana.del.rio@sergas.es (L.Q.-D.); Jorge.Suanzes.Hernandez@sergas.es (J.S.-H.); 2 Departamento de Ciencias da Saúde, Universidade da Coruña (UDC), 15001 A Coruña, Spain; maria.sobrido@udc.es; 3 CLINURSID Research Group, Psychiatry, Radiology, Public Health, Nursing and Medicine Department, Universidade de Santiago de Compostela, 15705 A Coruña, Spain; 4 Health Research Institute of Santiago, University Hospital of Santiago de Compostela-CHUS, 15706 A Coruña, Spain

**Keywords:** emergency medical services, blood gas analysis, nurses

## Abstract

Aim. To evaluate the influence of time on arterial blood gas values after artery puncture is performed. Method. Prospective longitudinal observational study carried out with gasometric samples from 86 patients, taken at different time intervals (0 (T0), 15 (T15), 30 (T30) and 60 (T60) min), from 21 October 2019 to 21 October 2020. The study variables were: partial pressure of carbon dioxide, bicarbonate, hematocrit, hemoglobin, potassium, lactic acid, pH, partial pressure of oxygen, saturation of oxygen, sodium and glucose. Results. The initial sample consisted of a total of 90 patients. Out of all the participants, four were discarded as they did not understand the purpose of the study; therefore, the total number of participants was 86, 51% of whom were men aged 72.59 on average (SD: 16.23). In the intra-group analysis, differences in PCO_2_, HCO_3_, hematocrit, Hb, K^+^ and and lactic acid were observed between the initial time of the test and the 15, 30 and 60 min intervals. In addition, changes in pH, pO_2_, SO_2_, Na and glucose were noted 30 min after the initial sample had been taken. Conclusions. The variation in the values, despite being significant, has no clinical relevance. Consequently, the recommendation continues to be the analysis of the GSA at the earliest point to ensure the highest reliability of the data and to provide the patient with the most appropriate treatment based on those results.

## 1. Introduction

Arterial gasometry (GA) is one of the most frequently used techniques in emergency clinical practice to assess respiratory function in patients due to its low cost, easy sampling, analysis and the possibility to check acid–base balance [1,2,3,4]

Even though scientific societies [1,5,6] present certain disagreements regarding sample gathering, there is unanimity in the fact that it should be analysed as soon as possible. For this reason, the sample must be sent to the laboratory in a container at 4 °C, in order to reduce the metabolism of the red blood cells. This metabolism will produce an increase in the value of pCO_2_ [7]. In emergency services, it is crucial to obtain quick results/rapid diagnosis. Peripheral devices for blood gas tests have been developed to speed up the process, so that there is no need to send samples to the laboratory.

The aim of the study was to evaluate the influence of time on arterial blood gas values after artery puncture is performed.

## 2. Materials and Methods

This was a prospective longitudinal observational study carried out with blood gas samples from 86 patients. All of them were asked by the research team to participate in the study by signing an informed consent.

The study was carried out in the emergency department of Hospital A Coruña from 21 October 2019 to 21 October 2020.

The inclusion criteria were individuals with a respiratory disease who were prescribed an arterial blood gas test by the medical personnel responsible for patients without life risk. Subjects with a positive Allen test, active fibrinolysis or suspected or active COVID-19 were excluded.

The study consisted of repeating the blood gas measurements of a single sample at different time intervals (0 (T0), 15 (T15), 30 (T30) and 60 (T60) min) with only the inclusion of the initial value in the clinical history. After 60 min, the samples were destroyed. The storage of the samples during the different time frames was carried out at room temperature (20–22 °C) and when the test was completed, they were shaken to homogenise them. The sample and analysis results were always collected by the same nurse. The materials used to perform the GSAs were plastic syringes (polypropylene) with a minimum filling volume of 1.5 cc of blood and the device with which the blood gases were measured was an ABL90 Flex Version 3.4

The variables under study were those provided by the gasometric assessment of partial pressure of carbon dioxide (PCO_2_), bicarbonate (HCO_3_), hematocrit, hemoglobin (Hb), potassium (K), lactic acid, pH, partial pressure of oxygen (PO_2_), saturation of oxygen (SO_2_), sodium (Na) and glucose. In addition, age and gender variables of the patients included in the study were collected.

The sample size was calculated based on the variation in the lactic acid values with an average difference between the first and the last measurement of 0.4 mmol/L, a standard deviation of 0.3 mmol/L, a confidence level of 95% and 97% power, and a minimum N of 25 patients was obtained. For the study of quantitative variables, normality was verified using the Kolmogorov–Smirnov test. The quantitative variables were expressed through measures of central tendency and dispersion as a function of normality (mean/median and standard deviation/interquartile range (IQR)) and the 95% confidence interval. To study the association between categorical variables, Pearson’s chi-square statistic and related variables were used, with the McNemar Test. The Friedman test was used for related samples and Bonferroni correction for the pairwise comparison. The data processing and analysis were carried out using the SPSS v.20.0 statistical package. A significance level of *p* < 0.05 was established.

## 3. Results

The initial sample consisted of a total of 90 patients. Two patients refused to participate in the study, one patient was discarded by the study investigators when it was suggested that the patient did not understand the information provided, and another patient was discarded because they did not have a family or legal representative at the time of their care in the emergency service department. As a result, the total number of participants was 86, 51% of whom were men; the mean age of study patients was 72.6 (SD: 16.23).

The data related to the gasometric values at the different times are shown in Table 1. Significant differences were observed in all variables in the intergroup analysis. The mean value decreased from T0 to T60 in pH, PCO_2_, PO_2_, HCO_3_, K and glucose; in contrast, an increase in the values of the variable SatO_2_ and lactic acid was evidenced.

In the intra-group analysis (Table 2), differences were observed in PCO_2_, HCO_3_^−^, hematocrit, Hb, K and lactic acid between the initial time and after 15, 30 and 60 min. In addition, changes in pH, pO_2_, SO_2_, Na and glucose were noted 30 min after the initial sample had been taken.

When assessing the normal values of the different variables, it was observed that those samples had increased in pH by 11.6% (T0: 54.7 vs. T60: 66.3%; *p* = 0.013) and glucose by 7% (T0: 24.4% vs. T60: 31.4%; *p* = 0.031) and lactic acid had increased significantly at all time frames (T0 vs. T15; *p* = 0.016, T0 vs. T30; *p* = 0.002 and T0 vs. T60; *p* <0.001).

## 4. Discussion

In our study, we observed how samples behave when stored at room temperature, the effects caused by the use of plastic syringes and a storage time during tests that exceeds all the recommendations of scientific societies.

Carrying out GSA in emergency services is the main diagnostic means of assessing a patient’s condition, therefore correct sampling and correct analysis are essential in order to avoid errors that have repercussions on patient safety.

Regarding the pH and pO_2_ values, we observed a decrease, as well as an increase in pCO_2_ after 60 min. These results differ from those obtained in the study by [8] carried out with a maximum time frame of 30 min, compared to our study that presents longer time intervals. The results are discordant even for 30 min tests. The disagreement between the pO_2_ values between both studies may be due to the fact that the oxygen input is greater in refrigerated plastic syringes compared to those that are stored at room temperature [7,9].

Variations in the increase in lactic acid as well as the decrease in glucose levels are explained by erythrocyte metabolism, which results in the formation of lactic acid, a decrease in pH and the formation of bicarbonate. Such marked variations in lactic acid levels are observed from minute 15 and are maintained over time. This continued increase over time is due to the non-refrigeration of the sample; when at room temperature, there is no slowing down of erythrocyte metabolism and, therefore, this increase occurs [10].

It is obvious that the variation in pO_2_, pCO_2_ and pH values is affected by the material of the extraction syringe, as well as the storage methods. These factors also influence lactic acid values, showing a clear rise over time.

Despite the variation and the existence of statistical significance, they does not correspond to clinical significance, as they remain within a range accepted as physiological, even though the storage is completely inappropriate. The usefulness of this study lies in the possibility of delaying the analysis of GA in high-stress situations, work overload or staff falling behind schedule, with lactic acid being the value that can cause the greatest change and lead to a wrong diagnosis. In our study, the main limitations are the changes in the sample collection and storage procedures, preventing us from comparing the results.

## 5. Conclusions

In this study, we can observe that although the samples were analysed under a totally discouraging preservation protocol, there are gasometric parameters with zero or minimal variations, as well as values with changes that, despite being significant, have no clinical relevance. The recommendation continues to be the analysis of GSA in the shortest time span to ensure the highest reliability of the data and provide the patient with the best treatment based on these results.

## Figures and Tables

**Table 1:** Gasometric values as a function of the analysis time since the extraction.

Parameter	Time				*p*-Value Inter-Groups	*p*-Value Intra-Groups
	T0 Mean (SD)	T15 Mean (SD)	T30 Mean (SD)	T60 Mean (SD)		
pH	7.41 (0.07)	7.41 (0.07)	7.40 (0.07)	7.39 (0.07)	**<0.001**	T0 vs. T15 = 0.101 **T0 vs. T30 < 0.001** **T0 vs. T60 < 0.001**
PCO_2_ mmhg	41.17 (10.51)	41.01 (10.34)	41.32 (10.45)	41.82 (10.24)	**<0.001**	**T0 vs. T15 < 0.001** **T0 vs. T30 < 0.001** **T0 vs. T60 < 0.001**
PO_2_ mmhg	81.37 (43.70)	81.80 (35.28)	81.94 (32.35)	80.82 (28.35)	**<0.001**	T0 vs. T15 = 1.000 **T0 vs. T30= 0.005** **T0 vs. T60 < 0.001**
HCO_3_ mmol/L	25.69 (4.84)	25.39 (4.79)	25.42 (4.82)	25.32 (4.72)	**<0.001**	**T0 vs. T15 < 0.001** **T0 vs. T30 < 0.001** **T0 vs. T60 < 0.001**
Hematocrit	39.38 (7.76)	39.47 (7.95)	40.62 (9.50)	40.50 (8.45)	**0.001**	**T0 vs. T15 = 0.019** **T0 vs. T30 = 0.012** **T0 vs. T60 = 0.003**
Hemoglobin g/dL	12.85 (2.53)	12.91 (2.63)	13.14 (3.37)	13.21 (2.76)	**0.004**	**T0 vs. T15 = 0.047** **T0 vs. T30 = 0.043** **T0 vs. T60 = 0.014**
SO_2_ %	93.31 (5.50)	104.15 (97.34)	114.42 (134.92)	93.77 (5.28)	**<0.001**	T0 vs. T15 = 1.000 T0 vs. T30 = 0.061 **T0 vs. T60 < 0.001**
K meq/L	4.19 (0.70)	4.13 (0.69)	4.11 (0.70)	4.08 (0.68)	**<0.001**	**T0 vs. T15 < 0.001** **T0 vs. T30 < 0.001** **T0 vs. T60 < 0.001**
Na meq/L	139.31 (3.61)	139.03 (3.72)	139.12 (3.58)	139.31 (3.76)	**<0.001**	T0 vs. T15 = 1.000 **T0 vs. T30 = 0.012** **T0 vs. T60 < 0.001**
Glucose mg/dL	147.16 (65.67)	147.53 (67.52)	144.12 (66.85)	140.50 (67.40)	**<0.001**	T0 vs. T15 = 0.101 **T0 vs. T30 < 0.001** **T0 vs. T60 < 0.001**
Lactic mmol/L	1.42 (1.00)	1.63 (1.02)	1.80 (1.02)	2.09 (1.05)	**<0.001**	**T0 vs. T15 < 0.001** **T0 vs. T30 < 0.001** **T0 vs. T60 < 0.001**

Bold: Statistically significant difference between groups: *p* < 0.05.

**Table 2:** Percentage of gasometric samples with normal values.

Parameter	Time				*p*-Value Intra-Groups
	0 Mean (SD)	15 Mean (SD)	30 Mean (SD)	60 Mean (SD)	
pH	54.7% (44.2–64.7%)	59.3% (48.7–69.1%)	61.6% (51.1–71.2%)	66.3% (55.8–75.4%)	T0 vs. T15 = 0.219 T0 vs. T30 = 0.700 T0 vs. T60 = 0.130
PCO_2_ mmhg	52.3% (41.9–62.6%)	52.3% (41.9–62.6%)	53.5% (43.0–63.7%)	55.8% (45.3–65.8%)	T0 vs. T15 = 1.000 T0 vs. T30 = 1.000 T0 vs. T60 = 0.453
PO_2_ mmhg	31.4% (22.6–41.8%)	34.9% (25.7–45.4%)	37.2% (27.7–47.8%)	38.4% (28.8–48.9%)	T0 vs. T15 = 0.250 T0 vs. T30 = 0.630 T0 vs. T60 = 0.700
HCO_3_ mmol/L	37.2% (27.7–47.8%)	34.9% (25.7–45.4%)	33.7% (24.6–44.2%)	32.6% (23.6–43.0%)	T0 vs. T15 = 0.723 T0 vs. T30 = 0.508 T0 vs. T60 = 0.289
Hematocrit %	52.3% (41.9–62.6%)	50.0% (39.7–60.3%)	46.5% (36.3–57.0%)	50.0% (39.7–60.3%)	T0 vs. T15 = 0.727 T0 vs. T30 = 0.302 T0 vs. T60 = 0.815
Hemoglobin g/dL	50.0% (39.7–60.3%)	51.2% (40.8–61.4%)	47.7% (37.4–58.1%)	51.2% (40.8–61.4%)	T0 vs. T15 = 1.000 T0 vs. T30 = 0.754 T0 vs. T60 = 1.000
SO_2_ %	81.4% (71.9–88.2%)	82.6% (73.2–89.1%)	82.6% (73.2–89.1%)	82.6% (73.2–89.1%)	T0 vs. T15 = 1.000 T0 vs. T30 = 1.000 T0 vs. T60 = 1.000
K meq/L	90.7% (82.7–95.2%)	90.7% (82.7–95.2%)	90.7% (82.7–95.2%)	90.7% (82.7–95.2%)	T0 vs. T15 = 1.000 T0 vs. T30 = 1.000 T0 vs. T60 = 1.000
Na meq/L	89.5% (81.3–94.4%)	83.7% (74.5–90.0%)	88.4% (79.9–93.6%)	88.4% (79.9–93.6%)	T0 vs. T15 = 0.125 T0 vs. T30 = 1.000 T0 vs. T60 = 1.000
Glucose mg/dL	24.4% (16.6–34.5%)	25.6% (17.5–35.7%)	30.2% (21.5–40.6%)	31.4% (22.6–41.8%)	T0 vs. T15 = 1.000 T0 vs. T30 = 0.063 **T0 vs. T60 = 0.031**
Lactic mmol/L	81.4% (71.9–88.2%)	73.3% (63.1–81.5%)	69.8% (59.4–78.5%)	54.7% (44.2–64.7%)	**T0 vs. T15 = 0.016** **T0 vs. T30 = 0.002** **T0 vs. T60 < 0.001**

Bold: Statistically significant difference between groups: *p* < 0.05.

## Data Availability

Data available upon request to the author, monteroenfermero@gmail.com.
